# Making ends meet – relating a self-reported indicator of financial hardship to health status

**DOI:** 10.1093/pubmed/fdad161

**Published:** 2023-08-24

**Authors:** Kate Homer, Jayne Taylor, Alexander Miller, Kate Pickett, Lucy Wilson, John Robson

**Affiliations:** Wolfson Institute of Population Health, Queen Mary University of London, London E1 2AB, UK; Hackney Council Department of Public Health, London E8 1DY, UK; United Kingdom Health Security Agency Nobel House, London SW1P 3JR, UK; University of York, Health Sciences, Heslington, York YO10 5DD, UK; Health Education England (East Midlands), St. Helen’s & Knowsley NHS Trust, St Helens WA9 3DA, UK; Wolfson Institute of Population Health, Queen Mary University of London, London E1 2AB, UK

**Keywords:** Finance, public health, social determinants

## Abstract

**Background:**

Area-based index of multiple deprivation (IMD) indicators of financial hardship lack individual specificity and sensitivity. This study compared self-reports of hardship with area measures in relation to health status.

**Methods:**

Interviews in one London Borough, reported financial hardship and health status. Associations of health status with most and least deprived quintiles of the IMD 2015 were compared with self-reported hardship; always or sometimes ‘having difficulty making ends meet at the end of the month’ in relation to never.

**Results:**

1024 interviews reported hardship status in 1001 (98%). 392 people (39%) reported they ‘always’ or ‘sometimes’ had hardship. In multivariate analysis, self-reported hardship was more strongly associated with smoking; odds ratio = 5.4 (95% CI: 2.8–10.4) compared with IMD, odds ratio = 1.9 (95% CI: 1.2–3.2). Health impairment was also more likely with self-reported hardship, odds ratio = 11.1 (95% CI: 4.9–25.4) compared with IMD; odds ratio = 2.7 (95% CI: 1.4–5.3). Depression was similarly related; odds ratio = 2.4 (95% CI: 1.0–5.6) and 2.7 (95% CI: 1.2–6.6), respectively.

**Conclusions:**

Self-reported hardship was more strongly related to health status than area-based indicators. Validity and implementation in routine health care settings remains to be established.

## Introduction

### Background

The Index of Multiple Deprivation (IMD) 2015 applies data from the UK census to geographical Lower Layer Super Output Areas (LSOAs) containing around 1500 residents. This composite score is based on 37 diverse indicators of deprivation averaged across all individuals living in that area. The IMD score is widely used for planning and research on health needs and inequalities.^1^ In primary care, IMD has been applied to individual patients based on their postcode for algorithms such as QRisk2 to inform treatment decisions, or more recently to identify people in more deprived population groups to prioritize care in the Covid pandemic.^2^^,^^3^ In addition, the socioeconomic context in which a person lives can influence health above and beyond their socioeconomic status at the individual level.^4^

However, individually reported measures may provide more granular information on the relationship between socioeconomic and health status. For mental health, objective socioeconomic indicators did not perform well in identifying poor mental wellbeing.^5^ People's self-reports of their social status are more strongly related to health than objective measures derived from administrative or area based sources.^6^

For ethnic minority populations, self-reported financial status was associated with positive social gradients for health outcomes, whereas objective measures of socioeconomic status were not.^7^

In urban areas, people from very different levels of affluence and poverty may live in close proximity and the use LSOA means that deprivation is averaged across all individuals living in the same area – levelling down the more affluent and levelling up the deprived, thus reducing the gradient of differences between the groups. Both in the UK and internationally, area level indicators have been shown to substantially underestimate individual levels of poverty and deprivation.^8–10^ Area-level as compared with individual-level socioeconomic indicators may also underestimate disease risk factors.^11^

In the context of direct health care provision, the client is a person with specific needs, and not an area with more general requirements. The IMD lacks specificity and sensitivity to reliably inform clinicians about financial hardship in individuals. In addition, IMD is updated every 10 years by the national Census information from which it is largely derived with four yearly partial updates. This further limits the utility of IMD as a contemporaneous measure of financial status or hardship. There has been much discussion therefore, about appropriate indicators to ‘screen for poverty’ and the identification of people, families and children, living in poverty, so that interventions can be more targeted and informed.^12–14^ There are clearly a wide range of potential indicators of financial hardship and in part the choice will depend on the purpose of such an indicator.^15^

The identification of poverty and financial hardship to determine its visibility, is an initial step on a longer pathway to actionable intervention both at an individual level and at aggregate levels to improve targeting of resources.^16^^,^^17^ Like ethnicity, if socioeconomic hardship goes unrecorded it will remain hidden in plain sight.

There are practical challenges and constraints about the information that can reasonably be obtained from individuals during routine health care delivery in general practice. Nevertheless, it is has been shown that it is possible to obtain, at national scale, high levels of coverage for self-reported indicators relating to inequalities, as indicated by the success of self-reporting and recording ethnic group among GP registered patients; averaging 70% nationally with recording >80% typical in ethnically diverse areas.^18^ This has involved a journey over 30 years from early adopters and enthusiasts in the 1990s to the current position of NHS mandated ethnicity recording for general practice introduced in 2020.^19^

There has been considerable debate about the most suitable measures that might indicate socioeconomic status and in particular, socioeconomic hardship.^20^ In the context of health, this has also included issues such as food insecurity and wellbeing.^21^^,^^22^ In the UK EPIC study, a simple self-report of current financial hardship (insufficient money for basic necessities) was associated with obesity even after adjusting for socioeconomic status.^23^

In Canada, primary care physicians developed a pragmatic question indicating financial hardship that is potentially feasible to be collected by primary care teams. They validated a self-report of socio-economic status as indicated by the response to the question; ‘Do you ever have difficulty making ends meet at the end of the month?’ with a four category response; ‘Never, Rarely, Sometimes or Always.’^24^ However, implementing these questions in routine primary care contexts presented challenges for the Canadian primary care teams in which it was piloted.^25^ In the UK, there have been calls for wider implementation of self-reports of financial hardship status. In the context of increasing financial hardship and health inequalities, the identification of financial hardship is an important data element, informing both direct care and planning.^26^

We report an initiative undertaken by Hackney Local Authority public health team, which aimed to determine the relationship between self-reported financial hardship and health indicators and contrast their relationship to the area-based IMD score.^27^

## Methods

### Data source

Data were taken from participants in the London Borough of Hackney, Health and Wellbeing survey in 2019. This study is a secondary analysis of data in this survey. We used three indicators commonly used as indicators of health status in primary care health records – smoking, physical health status and depression. The questionnaire contained further information on physical activity, alcohol use and nutrition but the questions were not in a format suitable for multivariate analysis.^27^

In the Health and Wellbeing survey, a random locational sampling approach was used, stratified by ward and local IMD 2015 quintile to direct the locations for face-to-face interviews with Hackney residents aged 16 years or older, during February and March 2019. Participants in the data collection areas were purposively selected to yield a quota of 10 interviews representative of the local population on the basis of age, gender, ethnic group, housing tenure and work status. Sampling continued until at least 1000 had responded to the questionnaire.^27^ Using English, the interviewers asked respondents for their responses to the questionnaire and the interviewer entered the responses.

Age-bands (16–24, 25–34, 35–44, 45–54, 55–64, 65–74, ≥75 years), ethnic group and sex were recorded for each respondent and grouped to approximate tertiles 16–34, 35–54 and ≥55 years, which reflect the relatively young age of the population in the borough.

Financial circumstance and hardship were defined by the ‘difficulty making ends meet’ question, ‘Do you ever have difficulty making ends meet at the end of the month?’ The respondents selected ‘Always,’ ‘Sometimes,’ ‘Rarely,’ ‘Never’ or ‘Preferred not to say.’ This measure of socio-economic status was compared with quintiles of the 2015 area based IMD score identified by respondents post-codes. This created five IMD deprivation groups. The following health indicators were collected from the respondents: current cigarette smokers; an average score for the Short Warwick-Edinburgh Mental Wellbeing Scale (SWEMWBS)^28^ and respondents who reported any physical or mental impairment or disability termed health impairment in this study. All dependent health outcomes were binary: current smokers (yes/no); having any physical or mental impairment or disability (Health impairment) (yes/no); and having a SWEMWBS score that indicated possible or probable depression (yes/no). The clinical cut-offs for SWEMWBS suggested by the authors divide the scores into high mental wellbeing (28–35); average mental wellbeing (21–27); possible depression (18–20) and probable depression (17 or less). We further collapsed the data for possible and probable depression versus high or average mental wellbeing into our health outcome of interest.^28^

### Statistical analysis

All statistical analyses were run in STATA MP 17.0. Initial analysis for the health outcomes compared the proportion of smokers, proportion of respondents with any health impairment and proportion of respondents with possible or probable depression in each category of IMD deprivation or self-reported financial circumstance measure using a Chi-squared test. A univariate logistic regression model was performed for each health outcome against each measure of deprivation or financial circumstance using IMD 2015 local quintiles and the four responses to the ‘difficulty making ends meet’ question. Odds ratios with confidence intervals were reported comparing the most and least deprived IMD quintile and those self-reporting hardship Always or Sometimes as compared with Never. Using a multivariate regression analysis, the model then adjusted for the potential confounders age-band, sex and ethnic group. A Hosmer-Lemeshow Chi-squared test was used to test goodness of fit.

## Results

### Population cohort

In the IMD 2015, Hackney was ranked among the 20 most deprived local authorities in England.^29^

Interviews were conducted with 1024 residents. Based on the 2017 Office for National Statistics mid-year population estimates for Hackney residents aged 16 and over, the sample size provided a 95% likelihood that the reported percentages were within 3.1% points of results reported had the whole population of Hackney had been surveyed.^27^ The sample population (Appendix Table 1) broadly reflected Hackney as a whole. 456 (44%) of the respondents were from Black and minority ethnic groups, and 780 (76%) of the survey population were under 55 years in keeping with Hackney’s ethnically diverse young population.^30^

### Responses to questionnaire

Of those responses to ‘Do you ever have difficulty making ends meet at the end of the month?’, 54 (5%) answered ‘Always’; 338 (33%), ‘Sometimes’ and 609 (60%) ‘Rarely’ or ‘Never.’ A further 23 respondents preferred not to answer the question (2%). For IMD local quintiles, similar numbers of respondents were in each quintile ranging from: 228 (22%) in the most deprived group to 189 (19%) in the least deprived quintile, reflecting the sampling strategy across the borough. (Appendix Table 1)

Appendix Table 1, describes responses to the health outcomes of interest in the survey. The number (proportion) of current smokers in the survey population was 206 (20%). The number of survey respondents with health impairment was 162 (16%).

The SWEMWBS mental health score for depression did not follow a normal distribution in the survey sample as would be expected in the general population.^28^ Of the total 953 answering SWEMWDS questions 874 (92%) had average or high mental wellbeing and 79 (8%) were identified with possible or probable depression.

### Univariate and multivariate analysis

**Fig. 1:**
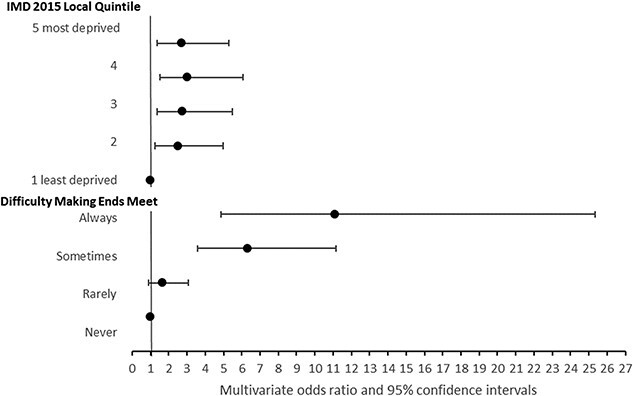
Reported smoking by area or self-reported economic indicator. Odds ratios adjusted for age band, sex and ethnic group.

**Fig. 2:**
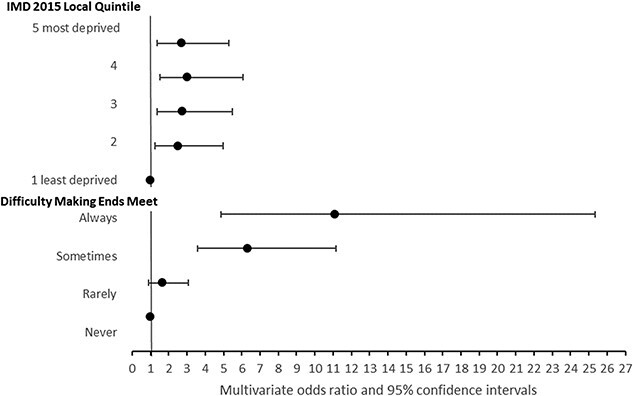
Reported disability by area or self-reported economic indicator. Odds ratios adjusted for age band, sex and ethnic group.

**Fig. 3:**
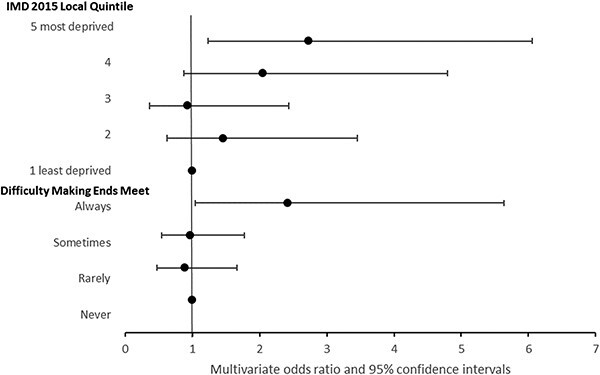
Reported depression by area or self-reported economic indicator. Odds ratios adjusted for age band, sex and ethnic group.

The univariate and multivariate analysis adjusting for age-band, sex and ethnic group showed a more pronounced association with self-reported health status using the ‘making ends meet’ question than IMD, with the exception of depression. Figures 1–3 show odds ratios for multivariate analyses with further detail in Appendix Tables 1 and 2. The Hosmer–Lemeshow test statistic was not significant for any of the multivariate models, indicating no evidence of poor fit.

In the univariate regression, for smoking, those who ‘Sometimes’ or ‘Always’ had ‘difficulty making ends meet’ were significantly more likely to smoke compared with those reporting ‘Never.’ People who ‘Always’ had ‘difficulty making ends meet’ were nearly four times as likely to smoke; odds ratio = 3.8 (95% CI: 2.0–7.0) and in multivariate analysis this increased to an odds ratio of 5.4 (95% CI: 2.8–10.4). (Figure 1, Appendix Table 2)

Using IMD quintile in the univariate regression, only the most deprived quintile showed any significant association with smoking; odds ratio = 1.6 (95% CI: 1.0–2.6) *P* = 0.06. In the multivariate analysis, this increased to 1.9 (95% CI: 1.2–3.2), *P* = 0.01 (Figure 1, Appendix Table 2).

Using ‘difficulty making ends meet’ in the univariate regression for health impairment, those who reported ‘Sometimes’ or ‘Always’ were significantly more likely to report health impairment compared with those reporting ‘Never’ having difficulty. People who ‘Always’ had difficulty were nearly 10 times as likely to report impairment, odds ratio = 9.5 (95% CI: 4.8–19.0), which was increased in multivariate analysis to 11.1 (95% CI: 4.9–25.4) (Figure 2, Appendix Table 2).

For IMD 2015 Quintile, the univariate regression of health impairment showed increased odds of impairment with increasing deprivation. Those respondents in the most deprived quintile reported more impairment compared with those in the least deprived quintile; odds ratio: 2.4 (95% CI: 1.3–4.5). In the multivariate regression, the odds ratio of impairment increased to 2.7 (95% CI: 1.4–5.3), *P* < 0.001 (Figure 2; Appendix Table 2).

For possible or probable depression, in univariate analysis, only those who ‘Always’ had ‘difficulty making ends meet’ were significantly more likely to report depression compared with those reporting ‘Never’ having difficulty with an odds ratio 2.7 (95% CI: 1.2–6.1). The multivariate analysis reduced the likelihood of depression for those who were ‘Always’ experiencing financial difficulty with an odds ratio of 2.4 (95% CI: 1.0–5.6) (Figure 3, Appendix Table 2).

Using the IMD quintiles, univariate regression showed odds of depression generally increasing with increasing deprivation. For respondents in the most deprived quintile the odds ratio was 2.6 (95% CI: 1.2–5.7) compared with the least deprived quintile. In the multivariate model, this increased to odds ratio 2.7 (95% CI: 1.2–6.6), *P* = 0.01 (Figure 3; Appendix Table 2).

## Discussion

### Main finding of this study

This study showed that the general population were willing to answer questions on financial hardship as indicated by the question ‘Do you ever have difficulty making ends meet at the end of the month?’ The association of health status as indicated by smoking and health impairment was more pronounced using a self-reported measure than with area based IMD status. This was particularly pronounced for those who reported they ‘always’ had ‘difficulty making ends meet’ with adjusted odds ratios for smoking in this group of 5.4 (95% CI: 2.8–10.4) compared with 1.9 (95% CI: 1.2–3.2) for IMD quintile 5 and for health impairment, odds ratio 9.5 (95% CI: 4.8–19.0) compared with IMD odds ratio 2.7 (95% CI: 1.4–5.3). For depression, the associations with the self-reports and IMD were similar.

### What is already known on this topic

The limitations of area based measures used in relation to individual health circumstances have been described earlier. The use of self-reported status has been highly effective in describing ethnic status in routine primary care settings in the UK and is now mandated for health providers.^19^ Self-reported financial hardship has been validated in Canada, though so far has not been scaled for use in routine health care settings.^24^^,^^25^ To date, the Canadian question ‘Do you ever have difficulty making ends meet at the end of the month?’ has not been economically validated in the UK and the association with health status has not been established.

### What this study adds

This study provides evidence that a question on financial hardship is acceptable to and answerable by almost all people in the general population. It confirms that self-reported financial hardship is more strongly associated with health status than area based IMD as indicated by smoking and health impairment. As in other studies, the relationship between depression and economic status is less pronounced.

### Limitations of this study

This study of self-reported health status was limited to three general health indicators that were available in the Wellbeing survey and are commonly recorded in primary care health records. For depression as assessed by a short questionnaire, ‘difficulty making ends meet’ did not add further information over IMD. The depression indicator derived from a short questionnaire may not represent clinical presentation and health service use. Other studies have found similar limitations of correlation between depression questionnaires and financial status.^5^

The population sample was representative of the Borough population; however, this was younger than the national average and more ethnically diverse. The proportion reporting health impairment was 16%, compared with 14.5% in the 2011 Census data for Hackney and the 20% proportion of current smokers in the survey population was higher than the 14% reported in the Annual Population Survey for 2019.^31^ Confidence intervals for many indicators were wide and overlapped for most of the odds ratios when comparing IMD with ‘difficulty making ends meet’. This reflects relatively small numbers with health conditions in the sample.

The questionnaire was administered by a company dedicated to the task, so that response rates might be higher than those obtained in routine settings without additional resources. This was the experience of a Canadian group who found the question difficult to implement in the setting of routine health care.^13^ The interviews were conducted in English, and this was not reported as a limitation.

While the case for individual level indicators of financial circumstance and hardship for use in routine health care setting is compelling, the validation of such measures as indicators of financial hardship remains to be established and would require detailed information on financial and social circumstance. The feasibility of implementing self-reported measures in routine service settings also needs to be established. Further research on both economic validation and implementation of the ‘difficulty making ends meet’ question would be useful contributions to the literature.

## Conclusion

A simple question on financial hardship was acceptable to the general population. Self-reported financial hardship was more strongly related to self-reported smoking and health impairment than area based IMD.

## Conflict of interest

JT, AM and LW were public health staff funded by the London Borough of Hackney. None of the authors have any interests to declare.

## Funding

The study was funded and Commissioned by the London Borough of Hackney as part of the 2019 Hackney Health and Wellbeing Survey conducted by BMG Research Ltd. Kate Pickett’s contribution was supported by the UK Prevention Research Partnership (MR/S037527/1) collaboration, ActEarly.

## Data availability

The original data from the Wellbeing Survey are not available.

## Authors’ contributions

JT and AM designed and commissioned the survey. JR and KH conducted the analysis, and all authors contributed to the manuscript. BMG Research Ltd conducted the interviews.

## Supplementary Material

Appendix_Tables_1_and_2_Ends_Meet_v10_fdad161

## Notes

**Kate Homer,** Analyst

**Jayne Taylor,** Consultant in Public Health

**Alexander Miller,** Senior Epidemiological Scientist

**Kate Pickett,** Professor of Epidemiology

**Lucy Wilson,** Public Health Analyst

**John Robson,** Reader in Primary Care
