# Correction to: Making ends meet – relating a self-reported indicator of financial hardship to health status

**DOI:** 10.1093/pubmed/fdad190

**Published:** 2023-09-08

This is a correction to: Kate Homer and others, Making ends meet – relating a self-reported indicator of financial hardship to health status, *Journal of Public Health*, 2023, fdad161, https://doi.org/10.1093/pubmed/fdad161

In the originally published version of this manuscript, Kate Pickett's funding information that underpinned her participation in the paper was omitted. Kate Pickett's contribution was supported by the UK Prevention Research Partnership (MR/S037527/1) collaboration, ActEarly.

This error has been corrected online.

