# Supplementary material for: Making ends meet – relating a self-reported indicator of financial hardship to health status
Source: J Public Health (Oxf). 2023 Aug 24;45(4):888–93. doi: 10.1093/pubmed/fdad161 (PMC10689002; doi:10.1093/pubmed/fdad161)
Supplement: Appendix_Tables_1_and_2_Ends_Meet_v10_fdad161 [file appendix_tables_1_and_2_ends_meet_v10_fdad161.doc]

**Appendix Table 1.**

**Respondent reporting of health status**

| **Health indicators** | **Number of respondents (%)** |
| --- | --- |
| **Current Smokers** | |
| Total responding  No | 1024  813 (79.4%) |
| Yes | 206 (20.1%) |
| Prefer not to say | 5 (0.5%) |
| **Reporting any health impairment** | |
| Total responding  No | 1024  850 (83.0%) |
| Yes | 162 (15.8%) |
| Prefer not to say | 12 (1.2%) |
| **Short Warwick-Edinburgh Mental Wellbeing Scale clinical cut-offs** | |
| Total responding  High mental wellbeing (28-35) | 1024  477 (46.6%) |
| Average mental wellbeing (21-27) | 397 (38.8%) |
| Possible depression (18-20) | 35 (3.4%) |
| Probable depression (17 or less) | 44 (4.3%) |
| Prefer not to say | 71 (6.9%) |

**Appendix Table 2.**

**Univariate and multivariate* Odds Ratios comparing ‘Difficulty Making Ends Meet’ with IMD 2015 local quintile for (A) current smokers, (B) health impairment (C) depression.**

| **A. SMOKING** | **Univariate** | | | | | **Multivariate ^a^** | | | |
| --- | --- | --- | --- | --- | --- | --- | --- | --- | --- |
|  | OR | LCI 95% | UCI 95% | P | | OR | LCI 95% | UCI 95% | P |
| **Difficulty Making Ends Meet** | | | | |  | | | | |
| Never | 1 (Ref) |  |  |  | | 1 (Ref) |  |  |  |
| Rarely | 1.13 | 0.74 | 1.73 | 0.56 | | 1.16 | 0.75 | 1.79 | 0.51 |
| Sometimes | 1.51 | 1.02 | 2.22 | 0.04 | | 1.59 | 1.06 | 2.38 | 0.03 |
| Always | 3.76 | 2.04 | 6.95 | 0.00 | | 5.41 | 2.81 | 10.43 | 0.00 |
| **IMD 2015 Local Quintile** | | | | | | | | | |
| 1 least deprived | 1 (Ref) |  |  |  | | 1 (Ref) |  |  |  |
| 2 | 1.26 | 0.77 | 2.07 | 0.36 | | 1.29 | 0.77 | 2.16 | 0.33 |
| 3 | 1.07 | 0.63 | 1.82 | 0.81 | | 1.21 | 0.70 | 2.08 | 0.50 |
| 4 | 1.25 | 0.74 | 2.11 | 0.40 | | 1.32 | 0.77 | 2.27 | 0.31 |
| 5 most deprived | 1.60 | 0.98 | 2.60 | 0.06 | | 1.91 | 1.15 | 3.17 | 0.01 |

| **B. HEALTH IMPAIRMENT** | OR | LCI 95% | UCI 95% | P | OR | LCI 95% | UCI 95% | P |
| --- | --- | --- | --- | --- | --- | --- | --- | --- |
| **Difficulty Making Ends Meet** | | | |  |  |  |  |  |
| Never | 1 (Ref) |  |  |  | 1 (Ref) |  |  |  |
| Rarely | 1.67 | 0.94 | 2.97 | 0.08 | 1.65 | 0.88 | 3.09 | 0.12 |
| Sometimes | 4.64 | 2.82 | 7.64 | 0.00 | 6.31 | 3.57 | 11.17 | 0.00 |
| Always | 9.50 | 4.75 | 19.02 | 0.00 | 11.12 | 4.88 | 25.36 | 0.00 |
| **IMD 2015 Local Quintile** | | | | | | | | |
| 1 least deprived | 1 (Ref) |  |  |  | 1 (Ref) |  |  |  |
| 2 | 2.00 | 1.07 | 3.74 | 0.03 | 2.50 | 1.26 | 4.98 | 0.01 |
| 3 | 2.33 | 1.24 | 4.38 | 0.01 | 2.73 | 1.36 | 5.47 | 0.01 |
| 4 | 2.47 | 1.31 | 4.66 | 0.01 | 3.01 | 1.50 | 6.07 | 0.00 |
| 5 most deprived | 2.44 | 1.32 | 4.50 | 0.00 | 2.69 | 1.36 | 5.30 | 0.00 |

| **C. DEPRESSION** | OR | LCI 95% | UCI 95% | P | OR | LCI 95% | UCI 95% | P |
| --- | --- | --- | --- | --- | --- | --- | --- | --- |
| **Difficulty Making Ends Meet** |  |  |  |  |  |  |  |  |
| Never | 1 (Ref) |  |  |  | 1 (Ref) |  |  |  |
| Rarely | 0.84 | 0.45 | 1.56 | 0.58 | 0.89 | 0.47 | 1.66 | 0.71 |
| Sometimes | 0.92 | 0.52 | 1.64 | 0.78 | 0.97 | 0.54 | 1.77 | 0.93 |
| Always | 2.74 | 1.23 | 6.13 | 0.01 | 2.42 | 1.04 | 5.64 | 0.04 |
| **IMD 2015 Local Quintile** | | | | | | | | |
| 1 least deprived | 1 (Ref) |  |  |  | 1 (Ref) |  |  |  |
| 2 | 1.34 | 0.57 | 3.15 | 0.50 | 1.46 | 0.62 | 3.45 | 0.39 |
| 3 | 0.94 | 0.36 | 2.43 | 0.90 | 0.93 | 0.36 | 2.43 | 0.88 |
| 4 | 2.12 | 0.94 | 4.93 | 0.07 | 2.05 | 0.87 | 4.80 | 0.10 |
| 5 most deprived | 2.61 | 1.20 | 5.69 | 0.02 | 2.73 | 1.23 | 6.06 | 0.01 |

*multivariate analysis by age-band, sex, ethnic group. Odds Ratios (OR) and Upper and Lower Confidence Intervals (UCI, LCI)
